# Promising drugs and treatment options for pediatric and adolescent patients with Hodgkin lymphoma

**DOI:** 10.3389/fcell.2022.965803

**Published:** 2022-11-24

**Authors:** Valli De Re, Ombretta Repetto, Lara Mussolin, Giulia Brisotto, Caterina Elia, Egesta Lopci, Emanuele S. G. d’Amore, Roberta Burnelli, Maurizio Mascarin

**Affiliations:** ^1^ Immunopatologia e Biomarcatori Oncologici, Dipartimento di Ricerca e Diagnostica Avanzata dei Tumori, CRO Aviano, National Cancer Institute, Istituto di Ricovero e Cura a Carattere Scientifico, IRCCS, Aviano, Italy; ^2^ Pediatric Hemato-Oncology Unit, Department of Women’s and Children’s Health, University of Padua, Padua, Italy; ^3^ AYA Oncology and Pediatric Radiotherapy Unit, Centro di Riferimento Oncologico IRCCS, Aviano, Italy; ^4^ Nuclear Medicine, IRCCS—Humanitas Research Hospital, Rozzano, MI, Italy; ^5^ Department of Pathology, San Bortolo Hospital, Vicenza, Italy; ^6^ Pediatric Hematology-Oncology Unit, Azienda Ospedaliera Universitaria, Ospedale Sant’Anna, Ferrara, Italy

**Keywords:** Hodgkin lymphoma, Epstein-Barr virus, chemotherapy, radiation, tumor target, adolescent

## Abstract

Currently-available therapies for newly-diagnosed pediatric and adolescent patients with Hodgkin lymphoma result in >95% survival at 5 years. Long-term survivors may suffer from long-term treatment-related side effects, however, so the past 20 years have seen clinical trials for children and adolescents with HL gradually abandon the regimens used in adults in an effort to improve this situation. Narrower-field radiotherapy can reduce long-term toxicity while maintaining good tumor control. Various risk-adapted chemo-radiotherapy strategies have been used. Early assessment of tumor response with interim positron emission tomography and/or measuring metabolic tumor volume has been used both to limit RT in patients with favorable characteristics and to adopt more aggressive therapies in patients with a poor response. Most classical Hodgkin’s lymphoma relapses occur within 3 years of initial treatment, while relapses occurring 5 years or more after diagnosis are rare. As the outcome for patients with relapsed/refractory classical Hodgkin lymphoma remains unsatisfactory, new drugs have been proposed for its prevention or treatment. This review summarizes the important advances made in recent years in the management of pediatric and adolescent with classical Hodgkin lymphoma, and the novel targeted treatments for relapsed and refractory classical Hodgkin lymphoma.

## Introduction: A model of Hodgkin lymphoma biology and biomarkers

Classical Hodgkin lymphoma (cHL) accounts for approximately 6%–7% of all pediatric cancers, with a peak incidence in adolescence and young adulthood. Reported incidence rates are: 29 myr in 15- to 19-year-olds; approximately 10/myr in 10- to 14-year-olds; 3.5/myr in children 5–9 years old; and 1/myr in infants up to 4 years old. Among Italian adolescents aged 15–19 years the incidence rate is 23.6/myr, which is twice the rate reported for the same age group in the United States and the rest of Europe (AIRTUM Working Group and CCM, 2013). HL is also associated with congenital immunodeficiency: it is estimated that 4.5% of HL cases are familial (McAulay and Jarrett, 2015).

There are four subtypes of cHL: nodular sclerosis (NSHL); mixed cell (MCHL); lymphocyte-rich (LRHL); and lymphocyte-depleted (LDHL) (Connors et al., 2020). NSHL is the most common of these subtypes. Patients with nodular lymphocyte-predominant HL (NLPHL) are classified as cases of non-cHL. Epstein-Barr virus (EBV) plays a role in the etiology of HL in approximately 30% of cases. The frequency of EBV positivity ranges from ∼75% in MCHL and LDHL to <20% in NSHL and LRHL (Massini et al., 2009).

**FIGURE 1:**
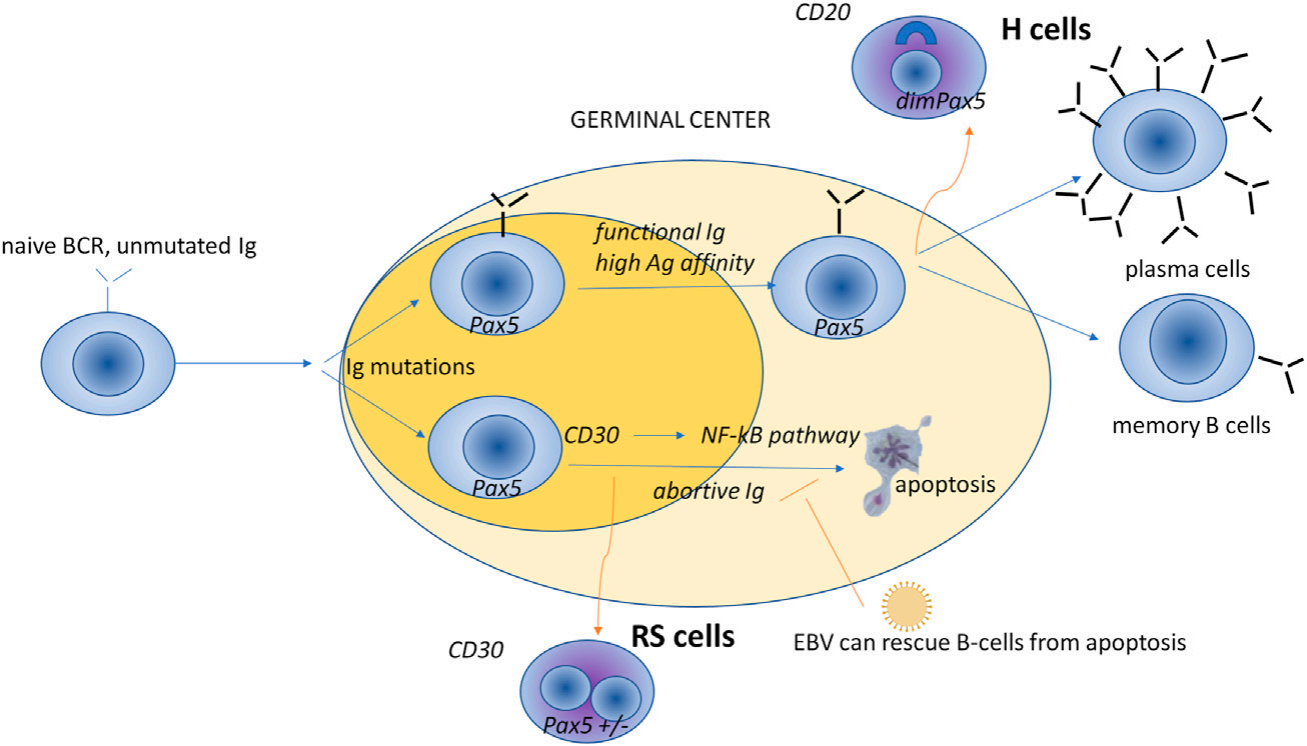
A model for the cellular germinal center origin of HRS cells. The origin of HRS cells has long been a matter of debate. Since the detection of clonal rearrangements of the immunoglobulin V gene in isolated HRS cells, it is now accepted that clonal B lymphocytes originate from HRS cells, with only a small proportion deriving from T lymphocytes. That said, HRS cells lose B-cell-specific genes (e.g., immunoglobulin genes and genes involved in the antigen presentation by MHC) and show characteristic positivity for nuclear paired box protein 5 (PAX5) (Khan et al., 2018) and CD30 antigen (Swerdlow et al., 2016; Weniger et al., 2018). PAX5 encodes a B-cell specific activator protein, a transcription factor expressed in the early but not in the late stages of B-cell differentiation. B-cell activation leads to the expression of the membrane receptor CD30, which regulates the apoptotic NF-kB pathway, and thus controls the load of the B-cell population (Küppers, 2009), B-cell receptor; dim, moderate expression; Hodgkin cells (H cells) of nodular lymphocyte-predominant HL; Ig, immunoglobulins; Reed-Sternberg (RS) cells of cHL.

Malignant cells are large and multinucleated, and derive from B lymphocytes. They are known as Hodgkin and Reed-Sternberg (HRS) cells. Figure 1 illustrates the model that the HRS cells originate from the postgerminal center. HRS cells are typically CD30^+^ and embedded in a tumor-promoting microenvironment (TME) rich in immune cells, where exhausted T cells, Th1 and Th2 T-helper (Th) cells, polarized regulatory T (T_reg_) cells, PD1+ T follicular helper (T_FH)_ cells, lymphocyte-activating 3 (LAG3, CD223)-positive T cells, and various subpopulations of macrophages play a key role in tumor support (for more details, see the next section). Patients with NLPHL have scattered, large neoplastic B cells with multilobate nuclei (lymphocyte-predominant or popcorn cells) expressing a broad panel of B-related markers (CD19, CD20, CD79a, PAX5, OCT2, BOB1) within nodules dominated by mantle zone B cells and follicular dendritic cells (FDCs) (Connors et al., 2020).

HL is diagnosed on histological examination of an excisional biopsy of a suspect lymph node. Though not specific for HRS cells when considered separately, the relevant immunohistochemical markers include the expression of CD15^+^ (Lewis X antigen), CD30^+^ (TNFRSF8), PAX5+ (B-cell transcription factor) with a typical loss of B-cell antigens (e.g., CD19, CD20, CD79a), surface membrane immunoglobulin (Ig), and some B-cell transcription factors (Oct2 and Bob1). In contrast, tumor cells in NLPHL retain a complete B-cell program (CD19^+^, CD20^+^, and all transcription factors), but are CD30- and CD15-negative. In cases of cHL associated with EBV infection, tumor cells express a type II latency pattern associated with EBV-encoded RNA (EBER), latent membrane proteins (LMP) 1 and 2a, EBV nuclear antigen 1 (EBNA 1), and BamH1-A right frame 1 (BARF1) (De Re et al., 2020).

The overexpression of novel markers in cHL has been investigated both for diagnostic purposes and for novel targeted therapies (for more details, see the following sections). These include: integrin-associated protein (IAP) CD47 (Lopez-Pereira et al., 2020); transcription factor GATA3 activated by the NF-kB pathway; signal transducer and activator of transcription (STAT) 6; cluster of differentiation (CD) 83; transcription factor FOXO3A; Wilms tumor gene-1 (WT1); preferentially expressed antigen in melanoma (PRAME); and survivin (Dave et al., 2022).

Extracellular vesicles (EVs) formed inside endosomal compartments (i.e., exosomes), and carrying RNA, DNA, and proteins, are actively released from HRS cells and function as intercellular messengers. In HL, serum EV levels increase considerably. The EVs are rich in CD30 antigen (Hansen et al., 2014), also known as tumor necrosis factor receptor 8 (TNFR8), and exhibit functional protumoral effects. Hansen et al. (2014) showed that CD30-rich EVs stimulate the secretion of the tumorigenic chemokine interleukin-8 (IL-8) by eosinophil-like cells and primary granulocytes. CD30-rich EVs also facilitate the crosstalk between HRS cells and immune cells in the microenvironment, thus playing a part in the recruitment of distant immune cells with a key role in tumor growth. A recently-developed diagnostic analytic technique involves an aurum nanoparticle (AuNPs) with peroxidase activity capable of binding and ascertaining the concentration of CD30 antigen in circulating EVs for the purpose of monitoring cHL activity (Slyusarenko et al., 2022).

EVs released by HL cells also carry high levels of the metallopeptidase ADAM10 (Tosetti et al., 2018). Using the ADAM10 inhibitors LT4 and CAM29 to counteract ADAM10 shedding from HRS cells was found to restore the expression on the HRS cell surface of the major histocompatibility complex (MHC) Class I chain-related protein A (MICA) (Tosetti et al., 2018), a ligand for the activating receptor NKG2D present on NK and cytotoxic CD8^+^ T cells; and this resulted in the activation of cytotoxic T cells. ADAM10 also targets CD30, leading to the release of soluble CD30, which could interfere with anti-CD30 treatments.

EVs were also found involved in the crosstalk between the HRS and fibroblasts, leading to the production of highly-activated cancer fibroblasts that contribute to the tumor-promoting microenvironment (TME) (Dörsam et al., 2018). Plasma EVs encode proteins such as fibrinogen *γ* chain, complement C4B (an isotype of C4 protein), and transthyretin, which are more abundant in pediatric relapsed HL (Repetto et al., 2021).

Many miRNAs, such as miR21-5p, miR127-3p, let7a-5p, miR24-3p, and miR155-5p, are strongly enriched in the EVs of patients with cHL. In addition, miR-155-5p is related to disease progression, and miR155 responds to chemotherapy (van Eijndhoven et al., 2016). In an *in vivo* model of EBV-positive cHL, HRS cells were shown to release the EBV-encoded miRNA BamHI fragment A rightward transcript (BART), which induces the expression of immunosuppressive IL-10, TNF-α, and arginase-1 in macrophages, further supporting the development of HL (Higuchi et al., 2018). HRS cells may also transfer cell membrane fragments—including important proteins such as PD-L1, CD38, CD137, and CD30—to non-cancerous cells, a phenomenon known as trogocytosis and indicated as another mechanism contributing to the establishment of a TME (Zeng and Schwarz, 2020).

## Genetic alterations in Hodgkin lymphoma

**FIGURE 2:**
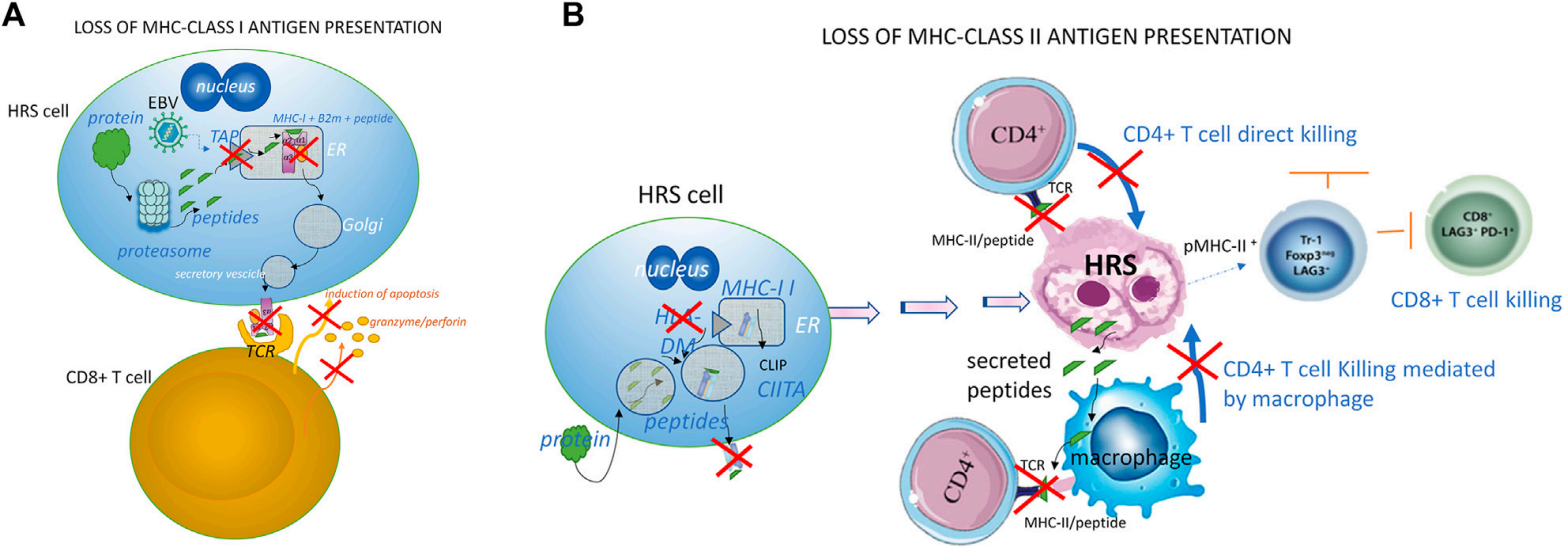
Loss of MHC class I **(A)** and class II **(B)** expression and MHC antigen presentation **(A)** A CD8^+^ cytotoxic T cell targets a cell expressing MHC-I/antigen complex after intracellular proteins have been digested by the proteasome into small peptides in Hodgkin and Reed-Sternberg cells, but the surface expression of the MHC-I/peptide complex is reduced due to transporters of peptides like processing (TAP) carriers and the beta 2 microglobulin (β2m) chain. Both the induction of apoptosis and the release of granzyme/perforin by CD8-T cells are consequently inhibited. In normal conditions, the peptides translocate into the endoplasmic reticulum (ER), allowing the antigen to bind to MHC-I. Then, after a passage in the Golgi structure and packaging in secretory vesicles, the MHC-I/peptide complex is fused with the plasma membrane and exposed to the surface of the target cell. The CD8^+^ T cell that recognizes and binds the MHC-I/peptide complex through its specific T-cell receptor (TCR) can induce the death of the target cell by activating the classical apoptotic signaling (Fas/caspase) pathway together with the release of the cytotoxic granzyme B and perforin from T-cell granules. In the case of tumor cells infected by EBV, the EBV BNLF2a, BGLF5, and BILF1 proteins produced during viral replication reduce the expression of MHC-I -antigen complexes, and thus contribute to the CD8^+^ T cell immune escape of the infected cells. **(B)** Direct and indirect killing of target cells by CD4^+^ T cells. MHC-II alpha and beta chains are normally assembled in the endoplasmic reticulum (ER). MHC-II binds the human leukocyte antigen DM (HLA-DM) to protect itself from endosomal degradation. HLA-DM releases the class II-associated invariant chain peptide (CLIP), replacing it with a sequence-specific peptide. The complex MHC-II/peptide (pMHC-II) goes to the cell surface, where MHC-II can present the peptide to the CD4^+^ T-cells. After the recognition of pMHC-II through its T-cell receptor (TCR), the CD4^+^ T-cell may kill the target cell directly by contact, or indirectly through the mediation of macrophages. Most HRS cells lack the HLA-DM, so the MHC-II is unprotected or unable to exchange the CLIP molecule with the antigenic peptide, and this leads to the absence of pMHC-II expression and a reduced tumor cell killing by CD4^+^ T-cells. The absence of pMHC-II expression is known to occur in about 40% of patients with cHL, and it is associated with poor treatment outcomes (Roemer et al., 2018). In other situations, HRS cells use pMHC-II to engage the immune suppressor lymphocyte-activation gene-3 (LAG3) receptor present on T cells, NK cells, plasmacytoid dendritic cells, and macrophages (Maruhashi et al., 2018; Triebel et al., 1990) in order to avoid immune recognition and exhaust cytotoxic T and NK cells. LAG-3 cooperates closely with PD-1 to alter regulator T cell homeostasis in cHL (Michot et al., 2021). Interestingly, CD4^+^ LAG3^+^ T cells have been found close to pMHC^+^ HRS cells, indicating that they play an important role in the development of the characteristic permissive microenvironment in cHL.

Patients have genetic alterations in their HRS cells (Table 1) that may also be present in their circulating mononuclear B cells, possibly due to telomere dysfunction (Buedts et al., 2021; Oki et al., 2015; M’kacher et al., 2018). The genetic basis for immune evasion and the anti-apoptotic signaling mediated by NF-κB and PI3K/AKT/mTOR pathways play a key role in cHL survival, while JAK/STAT signaling is known to sustain tumor growth. A reduced MHC Class I and/or Class II antigen expression and a reduced antigenic peptide/MHC complex presentation (Figure 2), which often correlates with worse prognosis, is reportedly common in HL, affecting from 40% to 70% of cases (Diepstra et al., 2007; Reichel et al., 2015).

**TABLE 1:** Known genetic aberrations in HRS cells.

Mechanism	Gene	Locus	Alteration	Function
Immune evasion	PDL1/2	Chr9p	Genomic amplification and rearrangements leading to overexpression	T-cell PD1/PDL immune checkpoint inhibition Roemer et al. (2016)
	B2M	Chr15q	Inactivating mutations	Limiting MHC Class I protein expression and associated antigen presentation to CD8 T cells Reichel et al. (2015) and Wienand et al. (2019)
	CIITA	Chr16p	Gene fusion	Limiting MHC Class II transcription and inducing immunosuppressive CD4^+^ LAG3+ (CD223) T cells Maruhashi et al. (2018)
	CD58	Chr1p	Truncating mutation/deletion	A ligand of the CD2 protein on T and NK cells, important for adhesion and activation of cytotoxic T and NK cells Schneider et al. (2015) and Veldman et al. (2020)
	CD47 (IAP)	Chr3q	Overexpression	CD47 interacts with SIRPα on macrophages, inhibiting phagocytic signals Lopez-Pereira et al. (2020)
Aberration related to pathway	JAK/STAT	JAK2, PDL, KDM4C, NOTCH1, Chr9p STAT3, STAT6, Chr12q SOCS1, Chr16p, PTPN-1, Chr20q Tiacci et al. (2018)	Copy number gains	Kinases that transport JAK/STAT signals are mainly produced by interleukin (IL) 4 and 13 from membrane receptors into the nucleus, where they activate genes of cell growth and induce PDL expression. KDM4C is a lysine demethylase, and a pro-oncogenic chromatin remodeler Shi et al. (2006) and Rui et al. (2010). It has also been reported that NOTCH activation can trigger NF-kB signaling, promoting the survival of cHL cells in cooperation with the EBV Schwarzer and Jundt (2011).
			Deletion/mutation	
	NF- κ B	JUNB (AP-1), Chr19p REL, exportin 1 (XPO1/CRM1), Chr2p Schwarzer and Jundt (2011), TNFAIP3, Chr6q Camus et al. (2016) and Desch et al. (2020)	Immunoglobulin gene translocation, copy number gains, mutation	They are positive regulators of NF- κB leading to B-cell survival and proliferation Gilmore et al. (2004) and Kober-Hasslacher and Schmidt-Supprian (2019). NF-κB controls JUNB Mathas et al. (2002).
	PI3K/Akt/mTOR	mTOR Chr1p	Constitutive high mTOR cell cycle activity	Key role in regulating cell functions such as survival, proliferation, cell death, and metabolic activities Feng et al. (2020) and Spina et al. (2018).
Family history	KLHDC8B	Chr3p	Loss of heterozygosity for actin-binding midbody protein expressed during cytokinesis	Promotion of the formation of the signature binucleated RS cells Salipante et al. (2009). Other genes are also found associated with altered cytokinesis in cHL Rengstl et al. (2013) and Webermatthiesen et al. (1995), (e.g., GNA13 and CDH1).
DNA methylation	Numerous B-cell transcription factors are downregulated, and transcriptional antagonists are upregulated		Several specific DNA methylation changes Wang et al. (2019b) and Ben Dhiab et al. (2015)	Methylated phenotype increases immune evasion
EBV infection	Latency II pattern generating intracellular EBNA1, viral-encoded RNAs EBER1/2, BART miRNAs, and the latent membrane proteins LMP1/2 (6) De Re et al. (2020)		Prevalence of EBV infection in less-developed nations, in immunodeficient and pediatric cases, and mixed cellularity and lymphocyte-depleted subtypes Dolcetti et al. (1995), Boiocchi et al. (1993), and Frisan et al. (1995)	Recovery of B cells from apoptosis

AP-1, activator protein-1; CD, cluster of differentiation; DNAm, DNA methylation; IAP, integrin-associated protein; Jak/STAT, Janus kinase/signal transducers and activators of transcription; mTOR, mechanistic target of rapamycin kinase; NF- κ B, nuclear factor kappa-light-chain-enhancer of activated B cells; PDL1(CD274), programmed cell death 1 ligand; PI3K/Akt, key regulator pathway of survival during cellular stress.

## Clinical studies for the treatment of young patients with Hodgkin lymphoma

The standard of care for adults with relapsed cHL involves high-dose chemotherapy combined with autologous stem cell transplantation after conventional chemotherapy. At the end of 2019, the EuroNet Group published guidelines for pediatric and adolescent patients with recurrent or refractory cHL based on risk stratification, response-adapted therapy, and a limited use of transplantation (Daw et al., 2020). It recommended initially stratifying patients in one of two risk groups (low- and standard-risk). At tumor relapse, the two groups were differentiated in terms of: 1) time to relapse; 2) tumor stage before first-line treatment; and 3) tumor stage and tumor burden at relapse. A third (high-risk) group included non-responders when positron emission tomography (PET) was positive (i.e., Deauville score 4, 5) after two cycles of salvage chemotherapy.

Patients in the low-risk group were treated with conventional-dose salvage therapy and RT. The standard-risk group received conventional-dose salvage chemotherapy plus consolidation with high-dose chemotherapy followed by autologous stem cell transplantation. Patients in the high-risk group were candidates for autologous or allogeneic transplantation, or experimental therapies. There is currently no gold standard for choosing the best first-line and rescue therapies. Clinical trials conducted in the 2000s using different combinations of conventional chemotherapy, alone or followed by RT, in children and adolescents with low-risk, intermediate/high-risk, or relapsed HL are listed in Tables 2–4, respectively.

**TABLE 2:** Main clinical trials in pediatric patients with low-risk HL.

Study groups	Period	Clinical studies	Pts (n)	Chemotherapy	Radiotherapy	ETS% (y)	OS (y)	
GPOH- HD95	1995–2001	CS IA/IB/IIA	328	2 O(P/E)PA	RC: no RT RP: 20–35Gy IF	PFS 93.2 (10)	98.8 (10)	Dörffel et al. (2013)
AIEOP MH96	1996–2004	CS IA, IIA no FPN*	159	3 ABVD	RC e PR >75%: 20Gy IF RP < 75%: 36Gy IF	92.8 (10)		Burnelli et al. (2018)
POG9426	1996–2000	CS I, IIA, and IIIA	294	DBVE x2-4 based on response after cycle 2	IF 25.5Gy IF	86.2 (8)	97.4 (8)	Tebbi et al. (2012)
GPOH-HD2002	2002–2005	CS IA/IB/IIA	195	F 2 OPPA M 2 OEPA	RC: no RT RP: 19.8Gy IF ± boost	93.2 (5) 91.7 (5)	100 (5) 100 (5)	Mauz-Körholz et al. (2010)
AIEOP MH2004	2004–2016	CS IA, IIA no FPN*	56	3 ABVD	RC no RT RP 25. 2Gy	95.8 (3)		Burnelli et al. (2020)
AHOD0431	2006–2012	CS IA and IIA HL without bulk	287	AVPC x3	CR after cycle 3: none RT; PR 21 RT IF	84 (10)	100 (2)	Keller et al. (2010)
EuroNet-PHL-C1	2007–2013	CS TG1: I A/B and II A without bulk ≥200 ml and without ESR ≥30 mm/h	714	OEPA x2	AR no RT IR RT 20Gy	90.6 (5)		Landman-Parker et al. (2016)

ABV, doxorubicin, bleomycin and vinblastine; ABVD, doxorubicin, bleomycin, vincristine, dacarbazine; ABVE-PC, doxorubicin, bleomycin, vincristine, etoposide, prednisone, cyclophosphamide; AIEOP, Associazione Italiana di Ematologia ed Oncologia Pediatrica; AVPC, doxorubicin, vincristine, prednisone, cyclophosphamide; COPP, cyclophosphamide, vincristine, procarbazine, prednisone; CS, clinical study; AR, adequate response; CR, complete remission; DFS, disease-free survival; DBVE, doxorubicin, bleomycin, vincristine, and etoposide; EFS, event-free survival; FPN, negative prognostic factors; FFP, freedom from progression; GPOH, German Pediatric Oncology Hematology; IF, involved-field radiotherapy. MOPP, mechlorethamine, vincristine, procarbazine, prednisone; IR, inadequate response; OPPA, vincristine, procarbazine, prednisone, doxorubicin; O(P/E)PA, vincristine (procarbazine or etoposide), prednisone, doxorubicin; OS, overall survival; PFS, progression-free survival; POG, Pediatric Oncology Group; RP, partial response; VAMP, vinblastine, doxorubicin (adriamycin), methotrexate, prednisone; VBVP, vinblastine, bleomycin, etoposide, prednisone.

**TABLE 3:** Main clinical trials on pediatric patients with intermediate- and high-risk HL.

Study groups	Period	Clinical stage	Pts (n)	Chemotherapy	Radiotherapy	ETS% (y)	DFS% (y)	OS% (y)	Reference
GPOH-HD95	1995–2001	CS IIB, IIIA, I/IIA_E_ CS, IIIB, IV, IIB_E_, IIIA/B_E_	256 341	2 OP (E) PA + 2COPP 2 OP (E) PA + 4COPP	RC: no RT RP: 20–35Gy IF RC: no RT RP: 20–35Gy IF		PFS 86.7 (10) PFS 84.5	97.3 (10) 93.2 (10)	Dörffel et al. (2013)
AIEOP MH96	1996–2004	CS IA, IIA con FPN*/IB, IIB, IIIA pts with M/T > 0.33, IIIB, IV	85 310	4 COPP/ABV 6 COPP/ABV	RC e RP >75% 20Gy IF RP <75%: 36Gy IF	80 (10) 75 (10)			Burnelli et al. (2018)
POG 9425	1997–2001	CS IB, IIA/IIIA_1,_ M/T>0.33, IIIA_2,_IIB/IIIB/IV	216	RER: 3 ABVE-PC SER: 5 ABVE-PC	21Gy IF 21Gy IF	86 (5) 83 (5)		95 (5) 95 (5)	Schwartz et al. (2009)
CCSG-59704	1999–2002	CS IIB/IIB w/bulk; IV	99	RER: BEACOPP +4 COPP/ABV (F) or 2 ABVD (M) SER: 8 BEACOPP	RER F: none RER M: 21–35Gy IF SER: 21–35Gy	94 (5)		97 (5)	Kelly et al. (2011)
GPOH-HD-2002	2002–2005	I_E_A/B, II_E_A, IIB, IIIA II_E_B, III_E_A/B, IIIB, IV	139 239	F 2 OPPA+2COPP M 2 OEPA+2COPDAC F 2 OPPA+4COPP M 2 OEPA+4COPDAC	19.8Gy IF	88.3 (5) 86.9 (5)	PFS 93.4 (5) 87.4 (5)	98.5 (5) 94.9 (5)	Mauz-Körholz et al. (2010)
AHOD0031	2002–2009	IB, IA/IIA with bulk, IIB, IIIA, IVA	1734	2ABVE-PC + RER:2ABVE-PC SER: 2ABVE-PC ± 2DECA	RER, CR: None RER, CR: 21 IF SER: 21 IF	83.5 (5)		97.3 (5)	Marks et al. (2018)
AIEOP LH2004	2004–2016	CS (G2) IA, IIA FPN*/IB, IIB, IIIA, (G3) pts with M/T > 0.33, IIIB, IV	48 152	4 COPP/ABV ± 2 IEP 6 COPP/ABV ± IEP	RC 14.4Gy RP 25.2Gy	82.8 (3) 78.7 (3)			Burnelli et al. (2020)
EuroNet-PHL-C1	2007–2013	CS: TG-2 (IAE, IBE, IIAE, II B or III A) and TG-3 (IIBE, IIIAE, IIIBE, III B or IV A and IVB)	1388	OEPA x2 + 2/4 COPP or 2/4 COPDAC	AR at ERA: no RT IR 20Gy + 10Gy boost if slow responder or residual volume >100 ml	TG2 93 (5) TG3 88 (5)			Mauz-Körholz et al. (2022)

ABV, doxorubicin, bleomycin, vinblastine; ABVD, doxorubicin (adriamycin), bleomycin, vincristine, dacarbazine; ABVE-PC, doxorubicin, bleomycin, vincristine, etoposide, cyclophosphamide; AIEOP, Italian Association of Hematology and Pediatric Oncology; AR, adequate response; BEACOPP, bleomycin, etoposide, doxorubicin, cyclophosphamide, vincristine, procarbazine, prednisone; CCSG, Children’s Cancer Study Group; CHOP, cyclophosphamide, doxorubicin, vincristine, prednisone; COP, cyclophosphamide, vincristine, procarbazine; COPDAC, cyclophosphamide, vincristine, prednisone, dacarbazine; COPP, cyclophosphamide, vincristine, procarbazine, prednisone; CS, clinical stage; DFS, disease-free survival; EF, extended fields radiotherapy; EFS, event-free survival; ERA, slow response to therapy; FFP, freedom from progression; IEP, isocyanide, etoposide, prednisone; IF, involved fields radiotherapy; GPOH, German Pediatric Oncology Hematology; IR, inadequate response; OEPA, vincristine, etoposide, prednisone, doxorubicin; O(P/E)PA, vincristine (procarbazine or etoposide), prednisone, doxorubicin; OPPA, vincristine, procarbazine, prednisone, doxorubicin; OS, overall survival; PFS, progression-free survival; PS, pathological stage (by exploratory laparotomy); pts, patients; POG, Pediatric Oncology Group; RC, complete remission; RER, rapid response to therapy; RP, partial response; VAMP, vinblastine, doxorubicin (adriamycin), methotrexate, prednisone; y, years. *FPN = negative prognostic factors.

**TABLE 4:** Response rate after standard chemotherapy in patients with relapsed HL.

Regime	Drugs	Patients (n)	ORR (%)	CR rate (%)	References
DHAP	Dexamethasone, high-dose Ara-C-cytarabine, cisplatin	102	88	21	Moskowitz et al. (2001)
IGEV	Ifosfamide, gemcitabine, vinorelbine, prednisolone	91	81	54	Baetz et al. (2003)
IEP-ABVD	Ifosfamide, etoposide, prednisolone, Adriamycin, bleomycin, vinblastine, dacarbazine	176	85	Not reported	Schellong et al. (2005)
ICE	Ifosfamide, carboplatin, etoposide	65	88	26	Santoro et al. (2007)
BeGEV	Bendamustine, gemcitabine, vinorelbine	59	83	73	Bartlett et al. (2007)
IVE	Ifosfamide, etoposide, epirubicin	51	84	60	Santoro et al. (2016)
GDP	Gemcitabine, dexamethasone, cisplatin	23	70	17	Younes et al. (2016)
GVD	Gemcitabine, vinorelbine, liposomal doxorubicin	91	70	19	Borchmann et al. (2017)

Consolidation RT improves progression-free survival in HL, but large irradiation fields are associated with an increased risk of secondary cancers and infertility, as well as heart and lung toxicities. Among these treatment-related complications, fertility issues have been paid particular attention in the setting of pediatric cancer care (given the patients’ young age), and fertility preservation is included in the discussion on the choice of treatment. In 2006, the American Society of Clinical Oncology (ASCO) published recommendations for fertility preservation in cancer patients. This document, and the update published in 2013, underscores the importance of discussing the risk of infertility associated with cancer therapies, and the options available for fertility preservation with patients as soon as possible. There are currently no effective ways to preserve fertility in prepubertal males with cancer, but most other patients can produce enough seminal fluid for cryopreservation before starting cancer therapy (Brannigan, 2014). Treatments may also adversely affect female reproductive organ function. In collaboration with the International Late Effects of Childhood Cancer Guideline Harmonization Group, the PanCareLIFE Consortium recently developed a practice guideline containing recommendations for fertility preservation in females with cancer up to 25 years of age (Mulder et al., 2021).

Fortunately, the use of smaller target volumes, combined with technological advances in treatment techniques, has led to the sparing of organs at risk without altering the efficient control of the tumor mass. Specifically, proton therapy has been proposed for treating mediastinal HL to significantly reduce the dose to organs at risk, such as cardiac substructures, and late toxicity (Loap et al., 2021). How to identify patients who may benefit from proton therapy is still being debated, however, as the slowness and complexity of technical aspects of the pretreatment setup and the cost of the instrument limit its routine use in clinical practice.

New target-specific biological drugs for cHL have been available since 2010, and have been used in combination with chemotherapy. Among them, brentuximab vedotin (Bv, SGN35), an anti-CD30 drug-conjugate antibody, and nivolumab, a monoclonal antibody (mAb) programmed cell death protein 1 (PD-1) inhibitor, were the first to be used in cases of relapsed/refractory cHL (Table 5). Clinical research on the specific clinical indications for their use, their association with traditional chemotherapy, and their potential long-term side effects is ongoing.

**TABLE 5:** New drugs and targeted treatments for children and young adults with cHL.

Drug	Class	Function	
Brentuximab vedotin (Bv, SGN35)	anti-CD30 drug-conjugate antibody	Antibody conjugated with the anti-mitotic agent monomethylastatin E (MMAE): it blocks the cell cycle and triggers apoptosis of HRS. Based on the promising results of a clinical trial Connors et al. (2018), the FDA approved Bv combined with chemotherapy for stage III-IV disease.	Cole et al. (2018), Traila et al. (2018), Castellino et al. (2019), and Wolska-Washer and Robak (2019)
Nivolumab Pembrolizumab	anti-programmed cell death protein 1 (PD-1) inhibitor	Inhibitor of PD-1/PD-L1 immune checkpoint	Cole et al. (2020), Davis et al. (2020), Kuruvilla et al. (2021), Sheikh et al. (2022), and Geoerger et al. (2020)
Promising new candidates			
Sintilimab, camrelizumab, tislelisumab	PD-1 inhibitor	Inhibitor of PD-1/PD-L1 immune checkpoint	Kaplon and Reichert (2019) and Song et al. (2021)
Ipilumumab	CTLA-4	Inhibitor of CTLA-4/CD80 and CD86 immune checkpoint	Diefenbach et al. (2020)
Anti-LAG-3	LAG-3 inhibitor	It reduces T-cell activation and mediates the immunosuppressive functions of regulatory cells	Nagasaki et al. (2020) and Tobin et al. (2021)
SRF231 TTI-621	anti-CD47 antibody	Inhibition of signal regulatory protein α (SIRPα)/CD47 interaction needed for macrophages and dendritic phagocytic activity	Peluso et al. (2020) and Ansell et al. (2021)
Anti-TIM-3	TIM-3 inhibitor	It inhibits cytotoxic T cells and Th1 responses, modulates T regulatory cell function, decreases monocyte/macrophage activation, and promotes expansion of myeloid-derived suppressor cells	El Halabi et al. (2021)
Car-T	T- cell based therapy	Chimeric antigen receptor modified T (Car-T) cell based therapy gives T lymphocytes the ability to combine both antigen recognition and T cell activation functions into a single receptor.	Bollard et al. (2014), Ramos et al. (2017), Ruella et al. (2017), Wang et al. (2017), Ramos et al. (2020), Gholiha et al. (2022), Meier et al. (2022), and Ho et al. (2021).
Trabectedin	CD30/CD37	A bispecific antibody targeting CD30 and CD37, both members of the tumor necrosis factor receptor family overexpressed on HRS cells: it increases HRS cell killing.	Casagrande et al. (2022)
90Y-daclizumab camidanlumab	anti-CD25 antibody	Human anti-CD25 antibody-radiolabeled or conjugated through a cathepsin-cleavable valine-alanine linker to a pyrrolobenzodiazepine (PBD) dimer toxin, SG3199.	Hamadani et al. (2021) and Janik et al. (2015)
Ruxolitinib Fedratinib Itacitinib	JAK inhibitors	Inhibitors of Janus kinases (JAK), selective for JAK1 and JAK2. A Phase I/II study (NCT03697408) of itacitinib in combination with everolimus in relapsed or refractory HL is ongoing.	Van den Neste et al. (2018)
Everolimus Idelalisib	mTOR inhibitor	Everolimus developed as an analog of rapamycin, an inhibitor of the serine protein kinase mTOR. Idelalisib, a PI3K inhibitor, reduces activation of the PI3K/Akt/mTOR pathway.	Johnston et al. (2018) and Gopal et al. (2017)
Selinexor	XPO1 inhibitor	Selective inhibitor of nuclear exportin 1 (XPO1) protein simultaneously inhibiting several active tumorigenic processes and also synergizing with other targeted drugs and chemotherapy Fisher et al. (2021). Hot spot mutations in HL Balasubramanian et al. (2022).	Galinski et al. (2021)
Vorinostat	HDAC inhibitors	Histone deacetylase (HDAC) inhibitors. Histones regulate gene expression and chromosome packaging during cell division.	Janku et al. (2021)
Decitabine	DNMT inhibitors	DNA methyltransferase (DNMT) inhibitors. In particular, EBV infection is associated with an altered expression of specific DNMTs Pei and Robertson (2020). A decreased expression of 5hmc in HRS is a common epigenetic marker Siref et al. (2020).	Falchi et al. (2016), Nie et al. (2019), and Wang et al. (2021)

Bv conjugated with the antimitotic agent monomethylastatin E (MMAE), a cell cycle blocker that leads to tumor cell apoptosis, targets the tumor necrosis factor CD30^+^ in RS cells. A Phase I/II trial conducted in Europe and the United States showed a good safety profile and clinical benefits in 36 children with relapsed/refractory cHL (Locatelli et al., 2018). This therapy can also bridge to curative autologous and/or allogeneic stem cell transplantation in patients initially considered transplant-ineligible. Reported adverse events include: neutropenia (11%); fever (6%); increased gamma-glutamyl transferase levels (6%); and peripheral neuropathy (33%). The Food and Drug Administration (FDA) and the European Medicines Agency (EMA) approved Bv for use in adult patients with cHL if stem cell transplantation fails, or in patients not candidates for transplantation after ≥2 cycles of chemotherapy. Current therapies, including high-dose chemotherapy and stem cell transplantation, cure approximately 50% of children and young people with relapsed/refractory chronic HL. The Center for International Blood and Marrow Transplant Research (CIBMTR) reported a progression-free survival rate of 56%, and an overall survival of 73% in 671 patients (age ≤30 years).

A combination of Bv and the chemotherapy drug bendamustine showed a promising activity in a Phase II study conducted on adults with relapsed/recurrent HL (O’Connor et al., 2018). When administered before autologous stem cell transplantation, the overall response rate was 93%, with a 74% complete response and a favorable safety profile. Authorization is needed for its use in children, however. More recently, brentuximab was used in combination with various chemotherapeutic agents, with or without RT, in pediatric and young adults with newly-diagnosed, advanced-stage HL (NCT02979522, NCT02166463, and NCT01920932); the results are under evaluation.

In 2016, the EMA approved the use of nivolumab, a PD-1/PD-L1 immune checkpoint inhibitor that regulates T lymphocyte function in the TME, for the treatment of adults with relapsed/refractory cHL after autologous stem cell transplantation and Bv consolidation. CheckMate 744 (NCT02927769) was the first Phase II trial involving nivolumab, Bv, and bendamustine with response-adapted therapy for children, adolescents, and young adults at low or standard risk of relapse. Preliminary results for the standard-risk cohort were presented by the American Society of Clinical Oncology (ASCO) in 2020 (Cole et al., 2020). Of the 44 patients enrolled in the study, 31 were children, and 17 of them had refractory disease while 14 had recurrent cHL. The treatment was well tolerated and resulted in a complete metabolic response rate of 88%, making it an effective rescue therapy for relapsed/refractory cHL before stem cell transplantation.

Combination treatments with Bv plus PD/PD-L1 inhibitors have reportedly been tried in an effort to avoid RT, but they can induce immune-related adverse events. Bv is associated with peripheral neuropathy or severe pulmonary toxicity when combined with bleomycin, and PD/PD-L1 inhibitors may cause autoimmune toxicities. Further investigations in randomized trials are needed to assess the benefits and best uses of these agents as frontline treatments. The results of the ongoing Phase III trial (NCT03907488) comparing Nivo-AVD with Bv-AVD are awaited with particular interest (Castellino et al., 2020), though their use in pediatric patients has yet to be formally authorized.

The safety and efficacy of pembrolizumab, another PD-1 inhibitor, are under evaluation in the MK-3475/KEYNOTE-667 trial involving children and young adults with newly-diagnosed cHL and an inadequate response to chemotherapy. The results will be compare with the findings of the standard EuroNet-PHL-C2 study. The widespread use of immunotherapy has made it difficult to assess patients using this new therapeutic modality accurately, however. As PET often fails to differentiate pseudoprogression from true tumor progression in patients treated with immune complex inhibitors (ICIs) (Cheson et al., 2016; Aide et al., 2019), new criteria are needed to help clinicians assess response to ICIs, such as the iRECIST guidelines (Persigehl et al., 2020). A small number of patients also continue to show resistance to these new treatments, and the results are unsatisfactory, so it remains important to explore new strategies and targets for treating cHL.

## Novel target drugs and treatment options in young patients with relapsed classic Hodgkin lymphoma

Table 5 gives a list of promising new targeted drugs. Alternative ICIs targeting PD-1/PD-L1 and other immune complexes that sustain an immunosuppressive TME have been discovered.

Sintilimab, camrelizumab, and tislelisumab all target PD-1. Ipilumumab targets the cytotoxic T lymphocyte-associated antigen-4 (CTLA-4) ligands CD80 (B7-1) and CD86 (B7-2), regulating the crosstalk between T cells and antigen-presenting cells (APC). Notably, CTLA-4+ cells, CD4^+^ Th1 polarized T cells, and T-reg (the main T-cell subsets in HL) are in close proximity to the CD68^+^ macrophages around HRS cells, and they are PD-1 negative (Patel et al., 2019). A Phase II trial is ongoing to verify the efficacy of dual PD-1 and CTLA-4 blockade with Bv (NCT01896999).

Relatlimab targets lymphocyte activation gene-3 (LAG-3; CD223 antigen). LAG-3 inhibits the function and expansion of both CD4 and CD8 T cells, serving as a marker of exhausted T cells during chronic antigen stimulation. LAG-3 is strongly expressed in T cells around the HRS cells, particularly in those lacking MHC-II expression (Gandhi et al., 2006), and in relapsed HL compared with cases in remission. The mechanism of T-cell inhibition is still poorly understood (Workman et al., 2002). The specific functional ligands of LAG-3 are still uncertain too, but it is now recognized that LAG-3, with its structural homology to the CD4 antigen (Triebel et al., 1990), preferentially recognizes specific MHC-II peptide complexes and consequently suppresses the function of specific CD4^+^ T cells (Maruhashi et al., 2022). Several other ligands for LAG-3 have been found, such as lymph node sinusoidal endothelial cell C-type lectin (LSECtin), galectin-3, and fibrinogen-like protein 1 (FGL1/FREP1), and their role in LAG3+ immune cell interactions is still under investigation (Chocarro et al., 2021; Wang et al., 2019).

SRF231 and TTI-621 block the interaction between CD47 (a “do not eat me” signal) and its ligand, the regulatory protein α (SIRPα), primarily expressed on phagocytic cells. Cases of cHL with a strong CD47 expression in HRS cells are associated with a shorter event-free survival (Ding et al., 2021; Gholiha et al., 2022).

More studies are needed on other immune checkpoint receptors that can downregulate immune cell function, such as T-cell immunoglobulin and mucin domain-containing protein 3 (TIM-3), T-cell immunoglobulin and ITIM domain (TIGIT), and V-domain Ig suppressor of T-cell activation (VISTA).

**FIGURE 3:**
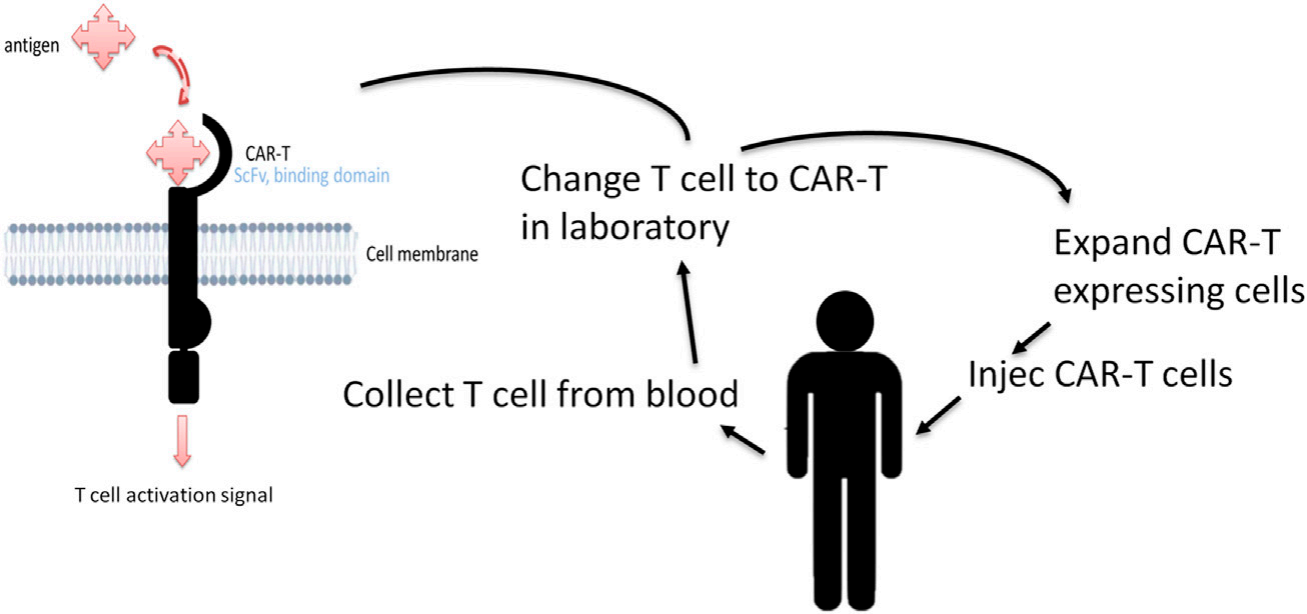
CAR-T structure and CAR-T cell therapy. The antigen-binding domain confers target specificity to CAR-T cells. This domain generally consists of variable heavy and light chains of monoclonal antibodies bound by a linker to form a single-chain variable fragment (scFv). The extracellular domain dictates the affinity and efficacy of the CAR-T cells and is independent of MHC-mediated antigen presentation. The difference in the intracellular co-stimulatory domains results in the modulation of different pathways like T cell differentiation and type of cell death. CAR-T treatment works by redirecting a patient’s own immune T cells, engineered in the laboratory, to directly identify and attack cancer cells.

In recent years, there has been increased interest in immunotherapies that use chimeric antigen receptor (CAR) T cells (Bollard et al., 2014; Ramos et al., 2017; Ruella et al., 2017; Wang et al., 2017; Ramos et al., 2020; Ho et al., 2021; Gholiha et al., 2022; Meier et al., 2022). Unlike conventional immunotherapies, CAR-T cells use engineered receptors that modulate the structure and function of human T-cell receptors (TCRs). CAR-T cells consist of an extracellular antigen-binding domain ahowing antigen specificity, a transmembrane domain, and one or more intracellular signaling domains (Figure 3). CAR-T cells packaged in a viral vector are then transported onto T cells to transduce CAR-T cells on the cell membrane surface. The T cells can thus recognize and kill the cells that express the target antigen. The power advantage of CAR-T cells over conventional immunotherapies lies in that they do not depend on antigenic peptide/MHC complex presentation for their activation, and in their long-term persistence and efficacy in the organism. Epitope location and antigen density on the surface of cancer cells may influence CAR-T cell efficiency. In addition, research into functional intracellular signaling domains has gained importance in the scientific community because intracellular co-stimulatory domains modulate several important cellular pathways, such as cell differentiation and cell death, which may increase CAR-T efficacy. Early studies demonstrating the advantage of CAR-T cell therapy in relapsed HL involved CAR-T cells directed against HRS cells expressing the EBV LMP-1 antigen (Bollard et al., 2014). Then CAR-T cells were used for all types of refractory/relapsed HL, regardless of their EBV status, by targeting HRS-specific surface markers such as CD30 molecules (Wang et al., 2017; Ramos et al., 2020). Several preclinical and clinical studies are now ongoing to upgrade the potential efficacy of CAR-T cell treatments using several approaches. The focus is mainly on targeting several antigens associated with HRS or immunosuppressive cell interactions in the TME, or on prolonging and increasing the immune response by increasing the living CAR-T cell levels and/or modulating the T-cell death pathway as recently reviewed in (Meier et al., 2022).

Other targetable drug strategies, in mono- or combined therapies, include targeting HRS cell markers using antibody-toxin conjugates such as camidanlumab, or specific molecular pathways that are altered in HL, such as JAK (i.e., ruxolitinib, fedratinib, itacinib), or using mTOR inhibitors (i.e., everolimus) (Table 5). Alternatively, CD30^−^ CD15^−^ peripheral B cells may be the progenitors of the CD30^+^ HRS cells (Jones et al., 2009), and thus B cells might be potential targets of therapy.

Another approach to treating HL involves potentiating the effects of conventional chemotherapies with the aid of nuclear export (XPOI) inhibitors (i.e., Selinexor), epigenetic histone deacetylases (HDACs), and DNA methyltransferases (DNMTs), for instance (Table 5). One promising strategy is based on the use of hypomethylating agents in combination with ICIs. The rationale behind this approach stems from the discovery that MHC gene silencing can be reversed using hypomethylating drugs, resulting in a greater efficacy of cytotoxic T cells according to several studies (Falchi et al., 2016; Nie et al., 2019; Wang et al., 2021). Since EBV latency is partly controlled by DNA methylation, however, the use of hypomethylating agents has only been proposed for the EBV-negative cHL subtype. The combination of HDAC and ICIs has also shown efficacy and adequate tolerability.

## Conclusion

Current research has led to the development of various strategies aimed at striking the best balance between supporting survival and reducing the long-term risk of toxicities related to therapies, including RT. The therapeutic strategies adopted for pediatric and young adults are consequently now quite different from those applied to adults and elderly patients with HL. It will be important to: 1) develop further studies on risk classification based not only on the classical parameters, but also using metabolic PET imaging; 2) identify patients eligible for proton therapy; and 3) discover new targeted drugs to include in more efficient combination treatment strategies. Studies on HRS cell biology and its close interaction with a multitude of cell subtypes in the TME could be useful for clarifying the molecular mechanisms that sustain the disease, and thereby reveal the best targets of treatment with a view to further reducing the risk of recurrence and long-term toxicity.

## Glossary

HL: Hodgkin lymphoma
cHL: classic Hodgkin lymphoma
RT: radiotherapy
PET: positron emission tomography
HRS: Hodgkin and Reed-Sternberg
LAG-3: lymphocyte activation gene 3
NLPHL: nodular lymphocyte-predominant HL
NSHL: nodular sclerosis Hodgkin lymphoma
MCHL: mixed cell Hodgkin lymphoma
LRHL: lymphocyte-rich Hodgkin lymphoma
LDHL: lymphocyte-depleted Hodgkin lymphoma
EBV: Epstein-Barr virus
EBER: EBV-encoded RNA
LMP: latent membrane protein
BCR: B-cell receptor
PAX5: nuclear-paired box protein 5
Ig: immunoglobulin
CD: cluster of differentiation
TNF: tumor necrosis factor
MHC: major histocompatibility complex
miRNA: microRNA
AP-1: activator protein-1
Jak/STAT: Janus kinase/signal transducers, and activators of transcription
mTOR: mechanistic target of rapamycin kinase
NF- κ B: nuclear factor kappa-light-chain-enhancer of activated B cells
PDL: programmed cell death ligand
TCR: T-cell receptor
mAb: monoclonal antibody
MMAE: anti-mitotic agent monomethylastatin E
Bv: Brentuximab vedotin
ICI: immune complex inhibitor
EMA: European Medicines Agency
SIRPα: signal regulatory protein α
TIM-3: T-cell immunoglobulin and mucin domain-containing protein 3
TIGIT: T-cell immunoglobulin and ITIM domain
VISTA: V-domain Ig suppressor of T-cell activation
HDAC: histone deacetylase
DNMT: DNA methyltransferase

## References

[B1] Aide N. Hicks R. J. Le Tourneau C. Lheureux S. Fanti S. Lopci E. (2019). FDG PET/CT for assessing tumour response to immunotherapy : Report on the EANM symposium on immune modulation and recent review of the literature. Eur. J. Nucl. Med. Mol. Imaging 46, 238–250. 10.1007/s00259-018-4171-4 30291373 PMC6267687

[B2] Airtum Working Group, Ccm (2013). Italian cancer figures, report 2012: Cancer in children and adolescents. Epidemiol. Prev. 37, 1–225. 23585445

[B3] Ansell S. M. Maris M. B. Lesokhin A. M. Chen R. W. Flinn I. W. Sawas A. (2021). Phase I study of the CD47 blocker TTI-621 in patients with relapsed or refractory hematologic malignancies. Clin. Cancer Res. 27, 2190–2199. 10.1158/1078-0432.CCR-20-3706 33451977

[B4] Baetz T. Belch A. Couban S. Imrie K. Yau J. Myers R. (2003). Gemcitabine, dexamethasone and cisplatin is an active and non-toxic chemotherapy regimen in relapsed or refractory Hodgkin’s disease: A phase II study by the national cancer institute of Canada clinical trials group. Ann. Oncol. 14, 1762–1767. 10.1093/annonc/mdg496 14630682

[B5] Balasubramanian S. K. Azmi A. S. Maciejewski J. (2022). Selective inhibition of nuclear export: A promising approach in the shifting treatment paradigms for hematological neoplasms. Leukemia 36, 601–612. 10.1038/s41375-021-01483-z 35091658 PMC8885406

[B6] Bartlett N. L. Niedzwiecki D. Johnson J. L. Friedberg J. W. Johnson K. B. van Besien K. (2007). Gemcitabine, vinorelbine, and pegylated liposomal doxorubicin (GVD), a salvage regimen in relapsed Hodgkin’s lymphoma: Calgb 59804. Ann. Oncol. 18, 1071–1079. 10.1093/annonc/mdm090 17426059

[B7] Ben Dhiab M. Ziadi S. Mestiri S. Ben Gacem R. Ksiaa F. Trimeche M. (2015). DNA methylation patterns in EBV-positive and EBV-negative Hodgkin lymphomas. Cell. Oncol. 38, 453–462. 10.1007/s13402-015-0242-8 PMC13004184 26350502

[B8] Boiocchi M. De Re V. Gloghini A. Vaccher E. Dolcetti R. Marzotto A. (1993). High-incidence of monoclonal ebv episomes in hodgkins-disease and anaplastic large-cell ki-1-positive lymphomas in hiv-1-positive patients. Int. J. Cancer 54, 53–59. 10.1002/ijc.2910540110 8386709

[B9] Bollard C. M. Gottschalk S. Torrano V. Diouf O. Ku S. Hazrat Y. (2014). Sustained complete responses in patients with lymphoma receiving autologous cytotoxic T lymphocytes targeting Epstein-Barr virus latent membrane proteins. J. Clin. Oncol. 32, 798–808. 10.1200/JCO.2013.51.5304 24344220 PMC3940538

[B10] Borchmann P. Goergen H. Kobe C. Lohri A. Greil R. Eichenauer D. A. (2017). PET-Guided treatment in patients with advanced-stage Hodgkin’s lymphoma (HD18): Final results of an open-label, international, randomised phase 3 trial by the German hodgkin study group. Lancet 390, 2790–2802. 10.1016/S0140-6736(17)32134-7 29061295

[B11] Brannigan R. E. (2014). Risk of infertility in male survivors of childhood cancer. Lancet. Oncol. 15, 1181–1182. 10.1016/S1470-2045(14)70450-4 25239574

[B12] Buedts L. Wlodarska I. Finalet-Ferreiro J. Gheysens O. Dehaspe L. Tousseyn T. (20212002). The landscape of copy number variations in classical hodgkin lymphoma: A joint KU leuven and LYSA study on cell-free DNA. Blood Adv. 5, 1991–2002. 10.1182/bloodadvances.2020003039 33843986 PMC8045498

[B13] Burnelli R. Fiumana G. Rondelli R. Pillon M. Sala A. Garaventa A. (2020). Comparison of Hodgkin’s lymphoma in children and adolescents. A twenty year experience with MH’96 and LH2004 AIEOP (Italian association of pediatric Hematology and Oncology) protocols. Cancers 12, 1620. 10.3390/cancers12061620 32570974 PMC7352443

[B14] Burnelli R. Rinieri S. Rondelli R. Todesco A. Bianchi M. Garaventa A. (2018). Long-term results of the AIEOP MH’96 childhood Hodgkin’s lymphoma trial and focus on significance of response to chemotherapy and its implication in low risk patients to avoid radiotherapy. Leuk. Lymphoma 59, 2612–2621. 10.1080/10428194.2018.1435872 29448858

[B15] Camus V. Stamatoullas A. Mareschal S. Viailly P-J. Sarafan-Vasseur N. Bohers E. (2016). Detection and prognostic value of recurrent exportin 1 mutations in tumor and cell-free circulating DNA of patients with classical Hodgkin lymphoma. Haematologica 101, 1094–1101. 10.3324/haematol.2016.145102 27479820 PMC5060026

[B16] Casagrande N. Borghese C. Aldinucci D. (2022). In classical Hodgkin lymphoma the combination of the CCR5 antagonist maraviroc with trabectedin synergizes, enhances DNA damage and decreases three-dimensional tumor-stroma heterospheroid viability. Haematologica 107, 287–291. 10.3324/haematol.2021.279389 34498447 PMC8719077

[B17] Castellino S. M. LeBlanc M. L. Herrera A. F. Parsons S. K. Punnett A. Hodgson D. C. (2020). An intergroup collaboration for advanced stage classical hodgkin lymphoma (cHL) in adolescents and young adults (AYA): Swog S1826. JCO 38, TPS8067. 10.1200/JCO.2020.38.15_suppl.TPS8067

[B18] Castellino S. M. Parsons S. K. Kelly K. M. (2019). Closing the survivorship gap in children and adolescents with Hodgkin lymphoma. Br. J. Haematol. 187, 573–587. 10.1111/bjh.16197 31566730

[B19] Cheson B. D. Ansell S. Schwartz L. Gordon L. I. Advani R. Jacene H. A. (2016). Refinement of the Lugano Classification lymphoma response criteria in the era of immunomodulatory therapy. Blood 128, 2489–2496. 10.1182/blood-2016-05-718528 27574190

[B20] Chocarro L. Blanco E. Zuazo M. Arasanz H. Bocanegra A. Fernández-Rubio L. (2021). Understanding LAG-3 signaling. Int. J. Mol. Sci. 22, 5282. 10.3390/ijms22105282 34067904 PMC8156499

[B21] Cole P. D. Mauz-Korholz C. Mascarin M. Michel G. Cooper S. Beishuizen A. Nivolumab and brentuximab vedotin (BV)-based, response-adapted treatment in children, adolescents, and young adults (CAYA) with standard-risk relapsed/refractory classical Hodgkin lymphoma (R/R cHL): Primary analysis. J. Clin. Oncol. (2020) 38: [Accessed April 19, 2022]8013, 10.1200/jco.2020.38.15_suppl.8013

[B22] Cole P. D. McCarten K. M. Pei Q. Spira M. Metzger M. L. Drachtman R. A. (2018). Brentuximab vedotin with gemcitabine for paediatric and young adult patients with relapsed or refractory Hodgkin’s lymphoma (AHOD1221): A children’s Oncology group, multicentre single-arm, phase 1-2 trial. Lancet. Oncol. 19, 1229–1238. 10.1016/S1470-2045(18)30426-1 30122620 PMC6487196

[B23] Connors J. M. Cozen W. Steidl C. Carbone A. Hoppe R. T. Flechtner H-H. (2020). Hodgkin lymphoma. Nat. Rev. Dis. Prim. 6, 61. 10.1038/s41572-020-0189-6 32703953

[B24] Connors J. M. Jurczak W. Straus D. J. Ansell S. M. Kim W. S. Gallamini A. (2018). Brentuximab vedotin with chemotherapy for stage III or IV Hodgkin’s lymphoma. N. Engl. J. Med. 378, 331–344. 10.1056/NEJMoa1708984 29224502 PMC5819601

[B25] Dave H. Terpilowski M. Mai M. Toner K. Grant M. Stanojevic M. (2022). Tumor-associated antigen-specific T cells with nivolumab are safe and persist *in vivo* in relapsed/refractory Hodgkin lymphoma. Blood Adv. 6, 473–485. 10.1182/bloodadvances.2021005343 34495306 PMC8791594

[B26] Davis K. L. Fox E. Merchant M. S. Reid J. M. Kudgus R. A. Liu X. (2020). Nivolumab in children and young adults with relapsed or refractory solid tumours or lymphoma (ADVL1412): A multicentre, open-label, single-arm, phase 1-2 trial. Lancet. Oncol. 21, 541–550. 10.1016/S1470-2045(20)30023-1 32192573 PMC7255545

[B27] Daw S. Hasenclever D. Mascarin M. Fernández-Teijeiro A. Balwierz W. Beishuizen A. (2020). Risk and response adapted treatment guidelines for managing first relapsed and refractory classical hodgkin lymphoma in children and young people. Recommendations from the EuroNet pediatric hodgkin lymphoma group. Hemasphere 4, e329. 10.1097/HS9.0000000000000329 32072145 PMC7000476

[B28] De Re V. Caggiari L. De Zorzi M. Fanotto V. Miolo G. Puglisi F. (2020). Epstein-Barr virus BART microRNAs in EBV- associated Hodgkin lymphoma and gastric cancer. Infect. Agent. Cancer 15, 42. 10.1186/s13027-020-00307-6 32582365 PMC7310352

[B29] Desch A-K. Hartung K. Botzen A. Brobeil A. Rummel M. Kurch L. (2020). Genotyping circulating tumor DNA of pediatric Hodgkin lymphoma. Leukemia 34, 151–166. 10.1038/s41375-019-0541-6 31431735

[B30] Diefenbach C. S. Hong F. Ambinder R. F. Cohen J. B. Robertson M. J. David K. A. (2020). Ipilimumab, nivolumab, and brentuximab vedotin combination therapies in patients with relapsed or refractory hodgkin lymphoma: Phase 1 results of an open-label, multicentre, phase 1/2 trial. Lancet. Haematol. 7, e660–e670. 10.1016/S2352-3026(20)30221-0 32853585 PMC7737486

[B31] Diepstra A. van Imhoff G. W. Karim-Kos H. E. van den Berg A. te Meerman G. J. Niens M. (2007). HLA class II expression by Hodgkin Reed-Sternberg cells is an independent prognostic factor in classical Hodgkin’s lymphoma. J. Clin. Oncol. 25, 3101–3108. 10.1200/JCO.2006.10.0917 17536082

[B32] Ding X-S. Mi L. Song Y-Q. Liu W-P. Yu H. Lin N-J. (2021). Relapsed/refractory classical hodgkin lymphoma effectively treated with low-dose decitabine plus tislelizumab: A case report. World J. Clin. Cases 9, 6041–6048. 10.12998/wjcc.v9.i21.6041 34368325 PMC8316945

[B33] Dolcetti R. Gloghini A. Devita S. Vaccher E. De Re V. Tirelli U. (1995). Characteristics of ebv-infected cells in hiv-related lymphadenopathy - implications for the pathogenesis of ebv-associated and ebv-unrelated lymphomas of hiv-seropositive individuals. Int. J. Cancer 63, 652–659. 10.1002/ijc.2910630509 7591281

[B34] Dörffel W. Rühl U. Lüders H. Claviez A. Albrecht M. Bökkerink J. (2013). Treatment of children and adolescents with hodgkin lymphoma without radiotherapy for patients in complete remission after chemotherapy: Final results of the multinational trial GPOH-HD95. J. Clin. Oncol. 31, 1562–1568. 10.1200/JCO.2012.45.3266 23509321

[B35] Dörsam B. Bösl T. Reiners K. S. Barnert S. Schubert R. Shatnyeva O. (2018). Hodgkin lymphoma-derived extracellular vesicles change the secretome of fibroblasts toward a CAF phenotype. Front. Immunol. 9, 1358. 10.3389/fimmu.2018.01358 29967610 PMC6015880

[B36] El Halabi L. Adam J. Gravelle P. Marty V. Danu A. Lazarovici J. (2021). Expression of the immune checkpoint regulators LAG-3 and TIM-3 in classical hodgkin lymphoma. Clin. Lymphoma Myeloma Leuk. 21, 257–266.e3. e3. 10.1016/j.clml.2020.11.009 33277223

[B37] Falchi L. Sawas A. Deng C. Amengual J. E. Colbourn D. S. Lichtenstein E. A. (2016). High rate of complete responses to immune checkpoint inhibitors in patients with relapsed or refractory Hodgkin lymphoma previously exposed to epigenetic therapy. J. Hematol. Oncol. 9, 132. 10.1186/s13045-016-0363-1 27899158 PMC5129196

[B38] Feng Y. Chen X. Cassady K. Zou Z. Yang S. Wang Z. (2020). The role of mTOR inhibitors in hematologic disease: From bench to bedside. Front. Oncol. 10, 611690. 10.3389/fonc.2020.611690 33489922 PMC7821787

[B39] Fisher J. G. Walker C. J. Doyle A. D. P. Johnson P. W. M. Forconi F. Cragg M. S. (2021). Selinexor enhances NK cell activation against malignant B cells via downregulation of HLA-E. Front. Oncol. 11, 785635. 10.3389/fonc.2021.785635 34926302 PMC8672299

[B40] Frisan T. Sjoberg J. Dolcetti R. Boiocchi M. De Re V. Carbone A. (1995). Local suppression of epstein-barr-virus (ebv)-Specific cytotoxicity in biopsies of ebv-positive hodgkins-disease. Blood 86, 1493–1501. 10.1182/blood.V86.4.1493.bloodjournal8641493 7632957

[B41] Galinski B. Alexander T. B. Mitchell D. A. Chatwin H. V. Awah C. Green A. L. (2021). Therapeutic targeting of exportin-1 in childhood cancer. Cancers 13, 6161. 10.3390/cancers13246161 34944778 PMC8699059

[B42] Gandhi M. K. Lambley E. Duraiswamy J. Dua U. Smith C. Elliott S. (2006). Expression of LAG-3 by tumor-infiltrating lymphocytes is coincident with the suppression of latent membrane antigen–specific CD8+ T-cell function in Hodgkin lymphoma patients. Blood 108, 2280–2289. 10.1182/blood-2006-04-015164 16757686

[B43] Geoerger B. Kang H. J. Yalon-Oren M. Marshall L. V. Vezina C. Pappo A. (2020). Pembrolizumab in paediatric patients with advanced melanoma or a PD-L1-positive, advanced, relapsed, or refractory solid tumour or lymphoma (KEYNOTE-051): Interim analysis of an open-label, single-arm, phase 1-2 trial. Lancet. Oncol. 21, 121–133. 10.1016/S1470-2045(19)30671-0 31812554

[B44] Gholiha A. R. Hollander P. Löf L. Glimelius I. Hedstrom G. Molin D. (2022). Checkpoint CD47 expression in classical Hodgkin lymphoma. Br. J. Haematol. 197, 580–589. 10.1111/bjh.18137 35301709 PMC9310712

[B45] Gilmore T. D. Kalaitzidis D. Liang M-C. Starczynowski D. T. (2004). The c-rel transcription factor and B-cell proliferation: A deal with the devil. Oncogene 23, 2275–2286. 10.1038/sj.onc.1207410 14755244

[B46] Gopal A. K. Fanale M. A. Moskowitz C. H. Shustov A. R. Mitra S. Ye W. (2017). Phase II study of idelalisib, a selective inhibitor of PI3Kδ, for relapsed/refractory classical Hodgkin lymphoma. Ann. Oncol. 28, 1057–1063. 10.1093/annonc/mdx028 28327905 PMC6246229

[B47] Hamadani M. Collins G. P. Caimi P. F. Samaniego F. Spira A. Davies A. (2021). Camidanlumab tesirine in patients with relapsed or refractory lymphoma: A phase 1, open-label, multicentre, dose-escalation, dose-expansion study. Lancet. Haematol. 8, e433–e445. 10.1016/S2352-3026(21)00103-4 34048682 PMC9241579

[B48] Hansen H. P. Engels H-M. Dams M. Paes Leme A. F. Pauletti B. A. Simhadri V. L. (2014). Protrusion-guided extracellular vesicles mediate CD30 trans-signalling in the microenvironment of Hodgkin’s lymphoma. J. Pathol. 232, 405–414. 10.1002/path.4306 24659185

[B49] Higuchi H. Yamakawa N. Imadome K-I. Yahata T. Kotaki R. Ogata J. (2018). Role of exosomes as a proinflammatory mediator in the development of EBV-associated lymphoma. Blood 131, 2552–2567. 10.1182/blood-2017-07-794529 29685921

[B50] Ho C. Ruella M. Levine B. L. Svoboda J. (2021). Adoptive T-cell therapy for Hodgkin lymphoma. Blood Adv. 5, 4291–4302. 10.1182/bloodadvances.2021005304 34610100 PMC8945637

[B51] Janik J. E. Morris J. C. O’Mahony D. Pittaluga S. Jaffe E. S. Redon C. E. (2015). 90Y-daclizumab, an anti-CD25 monoclonal antibody, provided responses in 50% of patients with relapsed Hodgkin’s lymphoma. Proc. Natl. Acad. Sci. U. S. A. 112, 13045–13050. 10.1073/pnas.1516107112 26438866 PMC4620907

[B52] Janku F. Park H. Call S. G. Madwani K. Oki Y. Subbiah V. (2021). Safety and efficacy of vorinostat plus sirolimus or everolimus in patients with relapsed refractory hodgkin lymphoma. Clin. Cancer Res. 26, 5579–5587. 10.1158/1078-0432.CCR-20-1215 33055173

[B53] Johnston P. B. Pinter-Brown L. C. Warsi G. White K. Ramchandren R. (2018). Phase 2 study of everolimus for relapsed or refractory classical Hodgkin lymphoma. Exp. Hematol. Oncol. 7, 12. 10.1186/s40164-018-0103-z 29774169 PMC5948762

[B54] Jones R. J. Gocke C. D. Kasamon Y. L. Miller C. B. Perkins B. Barber J. P. (2009). Circulating clonotypic B cells in classic Hodgkin lymphoma. Blood 113, 5920–5926. 10.1182/blood-2008-11-189688 19188663 PMC2700327

[B55] Kaplon H. Reichert J. M. (2019). Antibodies to watch in 2019. mAbs 11, 219–238. 10.1080/19420862.2018.1556465 30516432 PMC6380461

[B56] Keller F. G. Nachman J. Constine L. Thomson J. McCarten K. M. Chen L. (2010). A phase III study for the treatment of children and adolescents with newly diagnosed low risk hodgkin lymphoma (HL). Blood 116, 767. 10.1182/blood.V116.21.767.767 20484084

[B57] Kelly K. M. Sposto R. Hutchinson R. Massey V. McCarten K. Perkins S. (2011). BEACOPP chemotherapy is a highly effective regimen in children and adolescents with high-risk hodgkin lymphoma: A report from the children’s Oncology group. Blood 117, 2596–2603. 10.1182/blood-2010-05-285379 21079154 PMC3062352

[B58] Khan M. R. Ahmad A. Kayani N. Minhas K. (2018). Expression of PAX-5 in B cell hodgkin and non hodgkin lymphoma. Asian pac. J. Cancer Prev. 19, 3463–3466. 10.31557/APJCP.2018.19.12.3463 30583670 PMC6428548

[B59] Kober-Hasslacher M. Schmidt-Supprian M. (2019). The unsolved puzzle of c-rel in B cell lymphoma. Cancers 11, 941. 10.3390/cancers11070941 31277480 PMC6678315

[B60] Küppers R. (2009). The biology of Hodgkin’s lymphoma. Nat. Rev. Cancer 9, 15–27. 10.1038/nrc2542 19078975

[B61] Kuruvilla J. Ramchandren R. Santoro A. Paszkiewicz-Kozik E. Gasiorowski R. Johnson N. A. (2021). Pembrolizumab versus brentuximab vedotin in relapsed or refractory classical hodgkin lymphoma (KEYNOTE-204): An interim analysis of a multicentre, randomised, open-label, phase 3 study. Lancet. Oncol. 22, 512–524. 10.1016/S1470-2045(21)00005-X 33721562

[B62] Landman-Parker J. Wallace H. Hasenclever D. Balwierz W. Karlen J. Fossa A. (2016). First international inter-group study for classical hodgkin lymphoma in children and adolescents: euronet-phl-c1. report of the latest interim analysis. Haematologica 101, 35.

[B63] Loap P. De Marzi L. Mirandola A. Dendale R. Iannalfi A. Vitolo V. (2021). Development and implementation of proton therapy for hodgkin lymphoma: Challenges and perspectives. Cancers 13, 3744. 10.3390/cancers13153744 34359644 PMC8345082

[B64] Locatelli F. Mauz-Koerholz C. Neville K. Llort A. Beishuizen A. Daw S. (2018). Brentuximab vedotin for paediatric relapsed or refractory Hodgkin’s lymphoma and anaplastic large-cell lymphoma: A multicentre, open-label, phase 1/2 study. Lancet. Haematol. 5, E450–E461. 10.1016/S2352-3026(18)30153-4 30290902

[B65] Lopez-Pereira B. Fernandez-Velasco A. A. Fernandez-Vega I. Corte-Torres D. Quiros C. Villegas J. A. (2020). Expression of CD47 antigen in Reed-Sternberg cells as a new potential biomarker for classical Hodgkin lymphoma. Clin. Transl. Oncol. 22, 782–785. 10.1007/s12094-019-02171-2 31359339

[B66] Marks L. J. Pei Q. Bush R. Buxton A. Appel B. Kelly K. M. (2018). Outcomes in intermediate-risk pediatric lymphocyte-predominant hodgkin lymphoma: A report from the children’s Oncology group. Pediatr. Blood Cancer 65, e27375. 10.1002/pbc.27375 30277639 PMC6192844

[B67] Maruhashi T. Okazaki I-M. Sugiura D. Takahashi S. Maeda T. K. Shimizu K. (2018) LAG-3 inhibits the activation of CD4(+) T cells that recognize stable pMHCII through its conformation-dependent recognition of pMHCII. Nat. Immunol. 19:1415–1426. 10.1038/s41590-018-0217-9 30349037

[B68] Maruhashi T. Sugiura D. Okazaki I-M. Shimizu K. Maeda T. K. Ikubo J. Binding of LAG-3 to stable peptide-MHC class II limits T cell function and suppresses autoimmunity and anti-cancer immunity. Immunity (2022) 55:912–924.e8. 10.1016/j.immuni.2022.03.013 35413245

[B69] Massini G. Siemer D. Hohaus S. (2009). EBV in hodgkin lymphoma. Mediterr. J. Hematol. Infect. Dis. 1, e2009013. 10.4084/MJHID.2009.013 21416003 PMC3033177

[B70] Mathas S. Hinz M. Anagnostopoulos I. Krappmann D. Lietz A. Jundt F. (2002). Aberrantly expressed c-Jun and JunB are a hallmark of Hodgkin lymphoma cells, stimulate proliferation and synergize with NF-kappa B. EMBO J. 21, 4104–4113. 10.1093/emboj/cdf389 12145210 PMC126136

[B71] Mauz-Körholz C. Hasenclever D. Dörffel W. Ruschke K. Pelz T. Voigt A. (2010). Procarbazine-free OEPA-COPDAC chemotherapy in boys and standard OPPA-COPP in girls have comparable effectiveness in pediatric Hodgkin’s lymphoma: The GPOH-HD-2002 study. J. Clin. Oncol. 28, 3680–3686. 10.1200/JCO.2009.26.9381 20625128

[B129] Mauz-Körholz C. Landman-Parker J. Balwierz W. Ammann R. A. Anderson R. A. Attarbaschi A. (2022). Response-adapted omission of radiotherapy and comparison of consolidation chemotherapy in children and adolescents with intermediate-stage and advanced-stage classical Hodgkin lymphoma (EuroNet-PHL-C1): A titration study with an open-label, embedded, multinational, non-inferiority, randomised controlled trial. Lancet. Oncol. 23, 125–137. 10.1016/S1470-2045(21)00470-8 34895479 PMC8716340

[B72] McAulay K. A. Jarrett R. F. (2015). Human leukocyte antigens and genetic susceptibility to lymphoma. Tissue Antigens 86, 98–113. 10.1111/tan.12604 26189878

[B73] Meier J. A. Savoldo B. Grover N. S. (2022). The emerging role of CAR T cell therapy in relapsed/refractory hodgkin lymphoma. J. Pers. Med. 12, 197. 10.3390/jpm12020197 35207685 PMC8877886

[B74] Michot J-M. Mouraud S. Adam J. Lazarovici J. Bigenwald C. Rigaud C. (2021). CD8+ T lymphocytes immune depletion and LAG-3 overexpression in hodgkin lymphoma tumor microenvironment exposed to anti-PD-1 immunotherapy. Cancers 13, 5487. 10.3390/cancers13215487 34771650 PMC8582920

[B75] M’kacher R. Frenzel M. Al Jawhari M. Junker S. Cuceu C. Morat L. (2018). Establishment and characterization of a reliable xenograft model of hodgkin lymphoma suitable for the study of tumor origin and the design of new therapies. Cancers (Basel) 10, E414. 10.3390/cancers10110414 PMC6265845 30384446

[B76] Moskowitz C. H. Nimer S. D. Zelenetz A. D. Trippett T. Hedrick E. E. Filippa D. A. (2001). A 2-step comprehensive high-dose chemoradiotherapy second-line program for relapsed and refractory hodgkin disease: Analysis by intent to treat and development of a prognostic model. Blood 97, 616–623. 10.1182/blood.v97.3.616 11157476

[B77] Mulder R. L. Font-Gonzalez A. Green D. M. Loeffen E. A. H. Hudson M. M. Loonen J. (2021). Fertility preservation for male patients with childhood, adolescent, and young adult cancer: Recommendations from the PanCareLIFE Consortium and the international late effects of childhood cancer guideline harmonization group. Lancet. Oncol. 22, e57–e67. 10.1016/S1470-2045(20)30582-9 33539754

[B78] Nagasaki J. Togashi Y. Sugawara T. Itami M. Yamauchi N. Yuda J. (2020). The critical role of CD4+ T cells in PD-1 blockade against MHC-II-expressing tumors such as classic Hodgkin lymphoma. Blood Adv. 4, 4069–4082. 10.1182/bloodadvances.2020002098 32870971 PMC7479950

[B79] Nie J. Wang C. Liu Y. Yang Q. Mei Q. Dong L. (2019) Addition of low-dose decitabine to anti-PD-1 antibody camrelizumab in relapsed/refractory classical hodgkin lymphoma. J. Clin. Oncol. 37:1479–1489. 10.1200/JCO.18.02151 31039052

[B80] O’Connor O. A. Lue J. K. Sawas A. Amengual J. E. Deng C. Kalac M. (2018). Brentuximab vedotin plus bendamustine in relapsed or refractory Hodgkin’s lymphoma: An international, multicentre, single-arm, phase 1-2 trial. Lancet. Oncol. 19, 257–266. 10.1016/S1470-2045(17)30912-9 29276022 PMC9098158

[B81] Oki Y. Neelapu S. S. Fanale M. Kwak L. W. Fayad L. Rodriguez M. A. (2015). Detection of classical Hodgkin lymphoma specific sequence in peripheral blood using a next-generation sequencing approach. Br. J. Haematol. 169, 689–693. 10.1111/bjh.13349 25818067 PMC5279064

[B128] Patel SS Weirather JL Lipschitz M Lako A Chen P-H Griffin GK Armand P Shipp MA Rodig SJ. The microenvironmental niche in classic Hodgkin lymphoma is enriched for CTLA-4-positive T cells that are PD-1-negative. Blood (2019) 134:2059–2069. 10.1182/blood.2019002206 31697809 PMC7218752

[B82] Pei Y. Robertson E. S. (2020). The crosstalk of epigenetics and metabolism in herpesvirus infection. Viruses 12, E1377. 10.3390/v12121377 PMC7760534 33271926

[B83] Peluso M. O. Adam A. Armet C. M. Zhang L. O’Connor R. W. Lee B. H. (2020). The Fully human anti-CD47 antibody SRF231 exerts dual-mechanism antitumor activity via engagement of the activating receptor CD32a. J. Immunother. Cancer 8, e000413. 10.1136/jitc-2019-000413 32345627 PMC7213910

[B84] Persigehl T. Lennartz S. Schwartz L. H. (2020). iRECIST: how to do it. Cancer Imaging 20, 2. 10.1186/s40644-019-0281-x 31900236 PMC6942293

[B85] Ramos C. A. Ballard B. Zhang H. Dakhova O. Gee A. P. Mei Z. (2017). Clinical and immunological responses after CD30-specific chimeric antigen receptor-redirected lymphocytes. J. Clin. Invest. 127, 3462–3471. 10.1172/JCI94306 28805662 PMC5669573

[B86] Ramos C. A. Grover N. S. Beaven A. W. Lulla P. D. Wu M-F. Ivanova A. (2020). Anti-CD30 CAR-T cell therapy in relapsed and refractory hodgkin lymphoma. J. Clin. Oncol. 38, 3794–3804. 10.1200/JCO.20.01342 32701411 PMC7655020

[B87] Reichel J. Chadburn A. Rubinstein P. G. Giulino-Roth L. Tam W. Liu Y. (2015). Flow sorting and exome sequencing reveal the oncogenome of primary Hodgkin and Reed-Sternberg cells. Blood 125, 1061–1072. 10.1182/blood-2014-11-610436 25488972

[B88] Rengstl B. Newrzela S. Heinrich T. Weiser C. Thalheimer F. B. Schmid F. (2013). Incomplete cytokinesis and re-fusion of small mononucleated Hodgkin cells lead to giant multinucleated Reed-Sternberg cells. Proc. Natl. Acad. Sci. U. S. A. 110, 20729–20734. 10.1073/pnas.1312509110 24302766 PMC3870723

[B89] Repetto O. Lovisa F. Elia C. Enderle D. Romanato F. Buffardi S. (2021). Proteomic exploration of plasma exosomes and other small extracellular vesicles in pediatric hodgkin lymphoma: A potential source of biomarkers for relapse occurrence. Diagn. (Basel) 11, 917. 10.3390/diagnostics11060917 PMC8223799 34063765

[B90] Roemer M. G. M. Advani R. H. Ligon A. H. Natkunam Y. Redd R. A. Homer H. (2016). PD-L1 and PD-L2 genetic alterations define classical hodgkin lymphoma and predict outcome. J. Clin. Oncol. 34, 2690–2697. 10.1200/JCO.2016.66.4482 27069084 PMC5019753

[B91] Roemer M. G. M. Redd R. A. Cader F. Z. Pak C. J. Abdelrahman S. Ouyang J. (2018) Major histocompatibility complex class II and programmed death ligand 1 expression predict outcome after programmed death 1 blockade in classic hodgkin lymphoma. J. Clin. Oncol. 36, 942–950. 10.1200/JCO.2017.77.3994 29394125 PMC5877802

[B92] Ruella M. Klichinsky M. Kenderian S. S. Shestova O. Ziober A. Kraft D. O. (2017). Overcoming the immunosuppressive tumor microenvironment of Hodgkin lymphoma using chimeric antigen receptor T cells. Cancer Discov. 7, 1154–1167. 10.1158/2159-8290.CD-16-0850 28576927 PMC5628114

[B93] Rui L. Emre N. C. T. Kruhlak M. J. Chung H-J. Steidl C. Slack G. (2010). Cooperative epigenetic modulation by cancer amplicon genes. Cancer Cell 18, 590–605. 10.1016/j.ccr.2010.11.013 21156283 PMC3049192

[B94] Salipante S. J. Mealiffe M. E. Wechsler J. Krem M. M. Liu Y. Namkoong S. (2009). Mutations in a gene encoding a midbody kelch protein in familial and sporadic classical Hodgkin lymphoma lead to binucleated cells. Proc. Natl. Acad. Sci. U. S. A. 106, 14920–14925. 10.1073/pnas.0904231106 19706467 PMC2736436

[B95] Santoro A. Magagnoli M. Spina M. Pinotti G. Siracusano L. Michieli M. (2007). Ifosfamide, gemcitabine, and vinorelbine: A new induction regimen for refractory and relapsed Hodgkin’s lymphoma. Haematologica 92, 35–41. 10.3324/haematol.10661 17229633

[B96] Santoro A. Mazza R. Pulsoni A. Re A. Bonfichi M. Zilioli V. R. (2016). Bendamustine in combination with gemcitabine and vinorelbine is an effective regimen as induction chemotherapy before autologous stem-cell transplantation for relapsed or refractory hodgkin lymphoma: Final results of a multicenter phase II study. J. Clin. Oncol. 34, 3293–3299. 10.1200/JCO.2016.66.4466 27382096

[B97] Schellong G. Dörffel W. Claviez A. Körholz D. Mann G. Scheel-Walter H-G. (2005). Salvage therapy of progressive and recurrent Hodgkin’s disease: Results from a multicenter study of the pediatric DAL/GPOH-HD study group. J. Clin. Oncol. 23, 6181–6189. 10.1200/JCO.2005.07.930 16135485

[B98] Schneider M. Schneider S. Zühlke-Jenisch R. Klapper W. Sundström C. Hartmann S. (2015). Alterations of the CD58 gene in classical Hodgkin lymphoma. Genes Chromosom. Cancer 54, 638–645. 10.1002/gcc.22276 26194173

[B99] Schwartz C. L. Constine L. S. Villaluna D. London W. B. Hutchison R. E. Sposto R. (2009). A risk-adapted, response-based approach using ABVE-PC for children and adolescents with intermediate- and high-risk hodgkin lymphoma: The results of P9425. Blood 114, 2051–2059. 10.1182/blood-2008-10-184143 19584400 PMC2744567

[B100] Schwarzer R. Jundt F. (2011). Notch and NF-kappa B signaling pathways in the biology of classical hodgkin lymphoma. Curr. Mol. Med. 11, 236–245. 10.2174/156652411795243423 21375490

[B101] Sheikh I. Nunez C. McCall D. Roth M. Cuglievan B. (2022). Programmed cell death protein blockade with pembrolizumab for classical Hodgkin lymphoma after autologous stem cell transplantation in an adolescent patient. Pediatr. Blood Cancer 69, e29390. 10.1002/pbc.29390 35077016

[B102] Shi S. Calhoun H. C. Xia F. Li J. Le L. Li W. X. (2006). JAK signaling globally counteracts heterochromatic gene silencing. Nat. Genet. 38, 1071–1076. 10.1038/ng1860 16892059 PMC3092431

[B103] Siref A. McCormack C. Huang Q. Lim W. Alkan S. (2020). Diminished expression of 5hmc in Reed-Sternberg cells in classical Hodgkin lymphoma is a common epigenetic marker. Leuk. Res. 96, 106408. 10.1016/j.leukres.2020.106408 32659407

[B104] Slyusarenko M. Shalaev S. Valitova A. Zabegina L. Nikiforova N. Nazarova I. (2022). AuNP aptasensor for hodgkin lymphoma monitoring. Biosens. (Basel) 12, 23. 10.3390/bios12010023 PMC8774100 35049651

[B105] Song H. Liu X. Jiang L. Li F. Zhang R. Wang P. (2021). Current status and prospects of camrelizumab, A humanized antibody against programmed cell death receptor 1. Recent Pat. anticancer. Drug Discov. 16, 312–332. 10.2174/1574892816666210208231744 33563158

[B106] Spina V. Bruscaggin A. Cuccaro A. Martini M. Di Trani M. Forestieri G. (2018). Circulating tumor DNA reveals genetics, clonal evolution, and residual disease in classical Hodgkin lymphoma. Blood 131, 2413–2425. 10.1182/blood-2017-11-812073 29449275

[B107] Swerdlow S. H. Campo E. Pileri S. A. Lee Harris N. Stein H. Siebert R. (2016). The 2016 revision of the World Health Organization classification of lymphoid neoplasms. Blood 127, 2375–2390. 10.1182/blood-2016-01-643569 26980727 PMC4874220

[B108] Tebbi C. K. Mendenhall N. P. London W. B. Williams J. L. Hutchison R. E. Fitzgerald T. J. (2012). Response-dependent and reduced treatment in lower risk hodgkin lymphoma in children and adolescents, results of P9426: A report from the children’s Oncology group. Pediatr. Blood Cancer 59, 1259–1265. 10.1002/pbc.24279 22911615 PMC3468662

[B109] Tiacci E. Ladewig E. Schiavoni G. Penson A. Fortini E. Pettirossi V. (2018). Pervasive mutations of JAK-STAT pathway genes in classical Hodgkin lymphoma. Blood 131, 2454–2465. 10.1182/blood-2017-11-814913 29650799 PMC6634958

[B110] Tobin J. W. D. Bednarska K. Campbell A. Keane C. (2021). PD-1 and LAG-3 checkpoint blockade: Potential avenues for therapy in B-cell lymphoma. Cells 10, 1152. 10.3390/cells10051152 34068762 PMC8151045

[B111] Tosetti F. Venè R. Camodeca C. Nuti E. Rossello A. D’Arrigo C. (2018). Specific ADAM10 inhibitors localize in exosome-like vesicles released by Hodgkin lymphoma and stromal cells and prevent sheddase activity carried to bystander cells. Oncoimmunology 7, e1421889. 10.1080/2162402X.2017.1421889 29721369 PMC5927526

[B112] Traila A. Dima D. Achimas-Cadariu P. Micu R. (2018). Fertility preservation in Hodgkin’s lymphoma patients that undergo targeted molecular therapies: An important step forward from the chemotherapy era. Cancer Manag. Res. 10, 1517–1526. 10.2147/CMAR.S154819 29942153 PMC6005299

[B113] Triebel F. Jitsukawa S. Baixeras E. Roman-Roman S. Genevee C. Viegas-Pequignot E. (1990). LAG-3, a novel lymphocyte activation gene closely related to CD4. J. Exp. Med. 171, 1393–1405. 10.1084/jem.171.5.1393 1692078 PMC2187904

[B114] Van den Neste E. Andre M. Gastinne T. Stamatoullas A. Haioun C. Belhabri A. (2018). A phase II study of the oral JAK1/JAK2 inhibitor ruxolitinib in advanced relapsed/refractory Hodgkin lymphoma. Haematologica 103, 840–848. 10.3324/haematol.2017.180554 29351986 PMC5927969

[B115] van Eijndhoven M. A. Zijlstra J. M. Groenewegen N. J. Drees E. E. van Niele S. Baglio S. R. (2016). Plasma vesicle miRNAs for therapy response monitoring in Hodgkin lymphoma patients. JCI Insight 1, e89631. 10.1172/jci.insight.89631 27882350 PMC5111516

[B116] Veldman J. Visser L. Huberts-Kregel M. Muller N. Hepkema B. van den Berg A. (2020). Rosetting T cells in Hodgkin lymphoma are activated by immunological synapse components HLA class II and CD58. Blood 136, 2437–2441. 10.1182/blood.2020005546 32589698 PMC7685209

[B117] Wang C. Liu Y. Dong L. Li X. Yang Q. Brock M. V. (2021). Efficacy of decitabine plus anti-PD-1 camrelizumab in patients with hodgkin lymphoma who progressed or relapsed after PD-1 blockade monotherapy. Clin. Cancer Res. 27, 2782–2791. 10.1158/1078-0432.CCR-21-0133 33674274

[B118] Wang C-M. Wu Z-Q. Wang Y. Guo Y-L. Dai H-R. Wang X-H. (2017). Autologous T cells expressing CD30 chimeric antigen receptors for relapsed or refractory hodgkin lymphoma: An open-label phase I trial. Clin. Cancer Res. 23, 1156–1166. 10.1158/1078-0432.CCR-16-1365 27582488

[B119] Wang J. Sanmamed M. F. Datar I. Su T. T. Ji L. Sun J. (2019). Fibrinogen-like protein 1 is a major immune inhibitory ligand of LAG-3. Cell 176, 334–347. 10.1016/j.cell.2018.11.010 30580966 PMC6365968

[B120] Wang J. Van Den Berg D. Hwang A. E. Weisenberger D. Triche T. Nathwani B. N. (2019). DNA methylation patterns of adult survivors of adolescent/young adult Hodgkin lymphoma compared to their unaffected monozygotic twin. Leuk. Lymphoma 60, 1429–1437. 10.1080/10428194.2018.1533128 30668190 PMC7781082

[B121] Webermatthiesen K. Deerberg J. Poetsch M. Grote W. Schlegelberger B. (1995). Numerical chromosome aberrations are present within the CD30+ Hodgkin and Reed-Sternberg cells in 100% of analyzed cases of Hodgkin's disease [see comments]. Blood 86, 1464–1468. 10.1182/blood.V86.4.1464.bloodjournal8641464 7632954

[B122] Weniger M. A. Tiacci E. Schneider S. Arnolds J. Rüschenbaum S. Duppach J. (2018). Human CD30+ B cells represent a unique subset related to Hodgkin lymphoma cells. J. Clin. Invest. 128, 2996–3007. 10.1172/JCI95993 29889102 PMC6025985

[B123] Wienand K. Chapuy B. Stewart C. Dunford A. J. Wu D. Kim J. (2019). Genomic analyses of flow-sorted Hodgkin Reed-Sternberg cells reveal complementary mechanisms of immune evasion. Blood Adv. 3, 4065–4080. 10.1182/bloodadvances.2019001012 31816062 PMC6963251

[B124] Wolska-Washer A. Robak T. (2019). Safety and tolerability of antibody-drug conjugates in cancer. Drug Saf. 42, 295–314. 10.1007/s40264-018-0775-7 30649747 PMC6399172

[B125] Workman C. J. Dugger K. J. Vignaw D. a. A. (2002). Cutting edge: Molecular analysis of the negative regulatory function of lymphocyte activation gene-3. J. Immunol. 169, 5392–5395. 10.4049/jimmunol.169.10.5392 12421911

[B126] Younes A. Santoro A. Shipp M. Zinzani P. L. Timmerman J. M. Ansell S. (2016). Nivolumab for classical Hodgkin’s lymphoma after failure of both autologous stem-cell transplantation and brentuximab vedotin: A multicentre, multicohort, single-arm phase 2 trial. Lancet. Oncol. 17, 1283–1294. 10.1016/S1470-2045(16)30167-X 27451390 PMC5541855

[B127] Zeng Q. Schwarz H. (2020). The role of trogocytosis in immune surveillance of Hodgkin lymphoma. Oncoimmunology 9, 1781334. 10.1080/2162402X.2020.1781334 32934884 PMC7466850

